# DNA Adducts Formed by Aristolochic Acid Are Unique Biomarkers of Exposure and Explain the Initiation Phase of Upper Urothelial Cancer

**DOI:** 10.3390/ijms18102144

**Published:** 2017-10-14

**Authors:** Marie Stiborová, Volker M. Arlt, Heinz H. Schmeiser

**Affiliations:** 1 Department of Biochemistry, Faculty of Science, Charles University, Albertov 2030, CZ-12843 Prague 2, Czech Republic; 2 Analytical and Environmental Sciences Division, MRC-PHE Centre for Environment and Health, King’s College London, London SE1 9NH, UK; volker.arlt@kcl.ac.uk; 3 NIHR Health Protection Research Unit in Health Impact of Environmental Hazards at King’s College London in partnership with Public Health England, London SE1 9NH, UK; 4 Division of Radiopharmaceutical Chemistry, German Cancer Research Center (DKFZ), Im Neuenheimer Feld 280, 69120 Heidelberg, Germany; h.schmeiser@dkfz-heidelberg.de

**Keywords:** aristolochic acid, carcinogenicity, mutagenesis, nephrotoxicity, DNA adduct formation

## Abstract

Aristolochic acid (AA) is a plant alkaloid that causes aristolochic acid nephropathy (AAN) and Balkan endemic nephropathy (BEN), unique renal diseases frequently associated with upper urothelial cancer (UUC). This review summarizes the significance of AA-derived DNA adducts in the aetiology of UUC leading to specific A:T to T:A transversion mutations (mutational signature) in AAN/BEN-associated tumours, which are otherwise rare in individuals with UCC not exposed to AA. Therefore, such DNA damage produced by AA-DNA adducts is one rare example of the direct association of exposure and cancer development (UUC) in humans, confirming that the covalent binding of carcinogens to DNA is causally related to tumourigenesis. Although aristolochic acid I (AAI), the major component of the natural plant extract AA, might directly cause interstitial nephropathy, enzymatic activation of AAI to reactive intermediates capable of binding to DNA is a necessary step leading to the formation of AA-DNA adducts and subsequently AA-induced malignant transformation. Therefore, AA-DNA adducts can not only be utilized as biomarkers for the assessment of AA exposure and markers of AA-induced UUC, but also be used for the mechanistic evaluation of its enzymatic activation and detoxification. Differences in AA metabolism might be one of the reasons for an individual’s susceptibility in the multi-step process of AA carcinogenesis and studying associations between activities and/or polymorphisms of the enzymes metabolising AA is an important determinant to identify individuals having a high risk of developing AA-mediated UUC.

## 1. Introduction

Human exposures to chemicals with carcinogenic potency are considered to be connected with cancer development, predominantly when such exposures are frequent and long-term [1,2,3]. Several reviews conducted by Poirier [1,2,3] provided an overview on the carcinogenic nature of specific exposures to chemicals such as polycyclic aromatic hydrocarbons (PAHs) and aromatic amines which are considered to be associated with cancers in the human population for centuries. The carcinogenicity of these chemicals was also determined in studies employing animal models (for a review see [2,4].

The formation of covalent DNA adducts by carcinogens is considered to be one of the earliest steps in the initiation phase of cancer development [5,6]. The formation of these DNA adducts is dependent on the type of DNA bases and is influenced by the DNA sequence context. DNA adducts can be repaired at different rates depending on the fact whether they are located or not on the transcribed or non-transcribed strand of DNA, in addition to the phenomenon which nucleotide sequences is modified [7,8]. Some highly reactive genotoxic carcinogens are capable of directly interacting with DNA (e.g., alkylating agents) but most of them (e.g., many PAHs [9] or heterocyclic aromatic amines (HAAs) [10]) are chemically inert and require metabolic conversion before exerting their genotoxic properties [3,10,11,12,13]. The covalent binding of carcinogens to DNA, which is causally related to tumorigenesis, is now considered as a central dogma of chemical carcinogenesis. This belief is supported by various observations, such as the facts that: (i) the carcinogenic properties of many carcinogens is, as mentioned above, dependent upon their activation to reactive electrophilic derivatives, which react with nucleophilic sites within DNA; (ii) the extent of DNA adduct formation can often be correlated with the magnitude of carcinogenic responses; and (iii) mutations in certain tumor suppressor genes and the activation of several proto-oncogenes can be mediated by the interaction of carcinogens with DNA.

However, since humans are exposed not only to one but to a complex mixture of carcinogens, direct proofs of an association of exposure to the development of a specific cancer type are rare. The plant carcinogen aristolochic acid (AA) and the mycotoxin aflatoxin B1 [14,15] are two rare examples where a distinct environmental exposure is linked to tumour development in humans. This review focuses on AA and the significance of AA-specific DNA adducts as biomarkers of AA exposure and markers for the development of upper urothelial cancer (UUC) in AA-exposed individuals. The review also focuses on the mechanisms of AA enzymatic activation and detoxification which are critical determinants for AA-induced UUC development.

## 2. Aristolochic Acid

**Figure 1:**
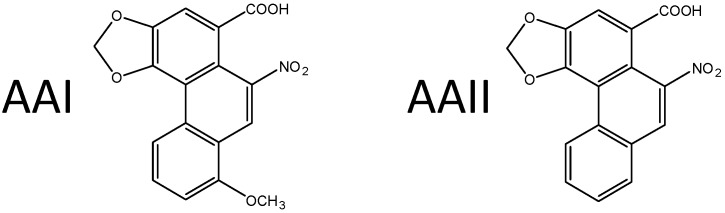
The major components of the AA plant extract, aristolochic acid I (AAI) and aristolochic acid II (AAII).

AA, the extract of plants of the Aristolochiaceae family, is a mixture of structurally related nitrophenanthrene carboxylic acids, with two major components: aristolochic acid I (8-methoxy-6-nitro-phenanthro-(3,4-*d*)-1,3-dioxolo-5-carboxylic acid, AAI) and aristolochic acid II (6-nitro-phenanthro-(3,4-*d*)-1,3-dioxolo-5-carboxylic acid, AAII) (Figure 1). AA is present particularly in plants of the *Aristolochia* and *Asarum* genera of the family Aristolochiaceae, in all plant parts. *Aristolochia* plants have been used for herbal medicinal remedies throughout the world since antiquity and they remain in use today, particularly in Chinese herbal medicine [16,17,18,19,20,21,22,23,24]. Both AAI and AAII are mutagenic and genotoxic [16,25,26,27,28] forming covalent DNA adducts after reductive activation in vitro and in vivo (reviewed in [16,17,18,19,20,21,22,23,24]).

### 2.1. Aristolochic Acid (AA) as a Carcinogen Causing Upper Urothelial Cancer in Aristolochic Acid Nephropathy (AAN) and Balkan Endemic Nephropathy (BEN) Patients and Renal Cell Carcinoma in Certain Other Human Populations

In 2012 AA was classified as carcinogenic to humans (Group 1) by the International Agency for Research on Cancer (IARC) acting by a genotoxic mechanism [29]. Today there is compelling evidence that human exposure to AA leads to UUC in patients with renal disease which is now termed aristolochic acid nephropathy (AAN) [29,30]. This disease is now recognized as a global disease; in Europe AAN has been found in Belgium, UK, France, Croatia, Serbia and Romania, in Asia in China and Taiwan [16,17,18,21,23,24,27,31,32,33].

The occurrence of this renal disease was first described by Vanherweghem et al. [33] and initially termed Chinese herbs nephropathy (CHN). Vanherweghem et al. [33] found this disease in young Belgian women treated with a sliming regimen in one clinic in Brussels. It was subsequently demonstrated that the pills used in the slimming regimen contained Chinese herbs that were contaminated with the nephrotoxin AA. Its presence in the slimming pills was the result of an accidental substitution of the prescribed herb *Stephania tetrandra* by *Aristolochia fangchi*, a plant species of the *Aristolochia* genus known to contain AA. Because AA was found to be the cause of this disease, it was later renamed as AAN [34,35]. Only a few years after the first description of AAN, UCC developed in almost 50% of these patients [27,36,37]. Recent studies indicated linking AA not only to upper urothelial cancers, but also renal cell carcinoma (RCC) [31] and to liver premalignant alterations [38,39].

AA is also considered as the major cause of another chronic renal disease associated with urothelial malignancy named Balkan endemic nephrophathy (BEN) [17,18,21,22,23,24,30,40]. It is believed that dietary contamination in endemic areas by *Aristolochia clematitis* seeds was the reason for AA exposure and subsequently the development of BEN. Indeed, a recent study showed that AAs could be released from the decay of *Aristolochia* plant growing abundantly as weeds in farmland in endemic areas of the Balkan Peninsula subsequently taken up by food crops from the polluted soil and contaminated the food grains [41].

#### 2.1.1. AA-Derived DNA Adducts and Their Role in the Initiation of Upper Urothelial Cancer

**Figure 2:**
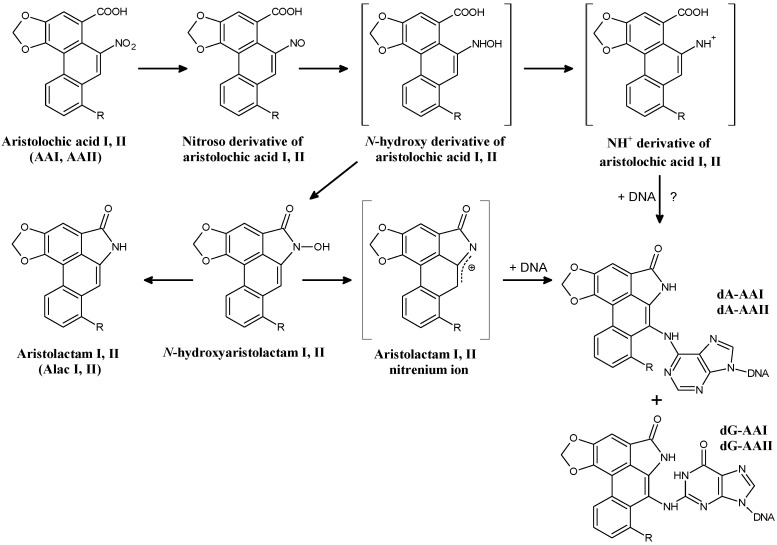
Reductive activation of aristolochic acid I [8-methoxy-6-nitro-phenanthro-(3,4-*d*)-1,3-dioxolo-5-carboxylic acid, AAI; R = OCH_3_] and II [6-nitro-phenanthro-(3,4-*d*)-1,3-dioxolo-5-carboxylic acid, AAII; R = H] leading to formation of DNA adducts. 7-(deoxyadenosin-*N*^6^-yl)aristolactam I or II (dA-AAI or dA-AAII), 7-(deoxyguanosin-*N*^2^-yl)aristolactam I or II (dG-AAI or dG-AAII). ?, possible pathway.

AAI and AAII are both enzymatically reduced to reactive cyclic acylnitrenium ions that can bind to the exocyclic amino groups of dA, dG and dC, forming covalent DNA adducts. In humans, specifically 7-(deoxyadenosin-*N*^6^-yl)aristolactam I or II (dA-AAI or dA-AAII) and 7-(deoxyguanosin-*N*^2^-yl)aristolactam I or II (dG-AAI or dG-AAII) have been identified (Figure 2) [17,18,42,43,44,45,46,47].

Specific AA-derived DNA adducts in renal and ureteric tissue of CHN/AAN patients were first determined by Schmeiser and coworkers [27,34,44,48,49], unequally proving exposure to AA in these patients. Furthermore, the detection of a specific AAG to TAG transversion mutation (an A:T to T:A transversion mutation) in the tumour suppressor gene *TP53* in one patient suffering from AAN-associated urothelial cancer [50] in combination with a high prevalence of A:T to T:A transversion mutations found in transgenic rodent mutation assays after AA exposure (reviewed in [50]) set the stage that this mutation pattern is now recognised as the mutational signature of AA and is used as another indicator for AA exposure [32,50]. Such A:T to T:A transversion mutations were later also confirmed in a larger group of CHN/AAN patients suffering from urothelial malignancy [51,52]. However, other types of mutations were also found in the tumours of these patients. The selectivity for mutations at adenine residues in AA-induced urothelial tumours corresponds to the high prevalence of dA-AAI adducts in the target tissue of CHN/AAN patients. This adduct exhibits a long persistence in renal tissue and is still detectable in CHN/AAN patients decades after AA exposure [53]. It was shown that mutated adenine found in *TP53* (i.e., codon 139) [49] has the same neighboring bases as in codon 61 (CAA) of the H-*ras* proto-oncogene in experimental animals (rats, mice). In these experimentally-induced tumours specific A:T to T:A transversions were also identified after AA treatment suggesting a sequence-specific mechanism during mutation induction [54,55,56,57,58]. Since the A:T to T:A transversions found in *TP53* correspond to the mutagenic specificity known for AA [54,55,56,57,58], it was proposed that these AA-induced transversion mutations in the *TP53* gene of urothelial tumours could be utilised as mechanistically relevant biomarkers of AA exposure in combination with specific AA-DNA adducts found in renal tissue of AAN patients [49,50]. This suggestion seems to be reasonable, because *TP53* mutations at these sites have not previously been associated with UUC and seem to be uniquely associated with exposure to AA [50,59,60]. Moreover, these findings explained the molecular mechanism, whereby AA causes urothelial malignancy [23,50].

The data showing that dA-AAI is the most abundant and persistent DNA adduct found in patients suffering from CHN/AAN [16,27,44,53,61,62] indicated that this adduct is an robust biomarker of AA exposure in patients suffering from this disease or in other individuals exposed to AA. AA-DNA adducts were also used as biomarkers of exposure in BEN patients. Exposure to AA in patients with renal disease living in areas endemic for BEN was first demonstrated by Arlt and collaborators [63] suggesting that AA is a causal factor in the development of BEN. Subsequently AA-DNA adducts in kidney tissue were proven in larger cohorts of patients with definite diagnosis of BEN, living in endemic regions in Croatia, Serbia, Bosnia and Romania [30,64,65]. Of note, no such AA-DNA adducts were identified in patients with other forms of chronic renal disease or patients with UCC living in non-endemic areas of Croatia and Serbia [64,65]. Similarly, Schmeiser and coworkers [30] determined AA-DNA adducts (i.e., dA-AAI) in kidney tissue of patients who underwent nephroureterectomy because of UUC and resided for 17 years or longer in villages of BEN areas in Romania. These results emphasise the significance of these DNA adducts and demonstrate their direct connections with AA exposures and with development of UUC in humans.

One important conclusion of all studies in CHN/AAN and BEN patients is that both diseases are preventable with simple control measures. Specifically, both diseases could be completely eliminated with a stronger regulation of herbal medicines and the prevention of dietary exposure to AA [23]. Even though herbal remedies containing AA have been banned in many countries, the risk of AA exposure caused by botanicals remains high in many regions of the world [23]. As a consequence, AAN can be considered, as it was postulated by Grollman [24], a global iatrogenic disease. From this point of view, an important advance in the ability to analyse AA-derived DNA adducts was recently achieved, because mass spectrometry has been proven to be a highly sensitive, specific and robust analytical method capable of identifying these adducts [53,66,67,68,69,70,71]. Mass spectrometry can therefore serve as an alternative to the ^32^P-postlabelling technique [64], the method which has been widely utilised over the last decades to detect and quantify AA-DNA adducts in human biomonitoring. Namely, mass spectrometry is able to identify the structure of the DNA adduct. The successful utilization of this approach was shown by analysing kidney tissue from Romanian cancer patients. RCC has not been reported in CHN/AAN patients previously but some Romanian patients unexpectedly showed high frequencies of A:T to T:A transversion mutations by whole-genome sequencing of the renal tumours, which is consistent with AA exposure [70]. A subsequent study utilised mass spectrometry and dA-AAI adducts were found in these Romanian cases unequivocally demonstrating exposure to AA in these patients [71]. As these patients do not cover the Romanian population of the BEN area [65], the source of AA exposure has not yet been identified in this cohort. Nevertheless, utilising AA-DNA adducts as biomarker of exposure and the unique mutational signature of AA as biomarker of effect clearly identified AA as an aetiologic agent of renal cell carcinoma seen in these patients. Indeed, recently Hoang et al. [31] also detected AA-derived DNA adducts in the kidneys of Taiwanese patients suffering from renal cell carcinoma providing additional evidence that AA not only causes UUC but also RCC.

The detection of AA-DNA adducts (i.e., dA-AAI) in the renal tissue of human individuals exposed to AA in several countries over the world is summarised in Table 1.

Besides demonstrating AA exposure by the detection of specific AA-DNA adducts, other approaches used the detection of A:T to T:A mutations in *TP53* as a marker of AA-induced cancer risk in Taiwan [72,73]. This study confirmed the hypothesis that the mutational signature of AA in the *TP53* gene found in UUC-associated with CHN/AAN or BEN [59,64], is the same as that determined in Taiwanese patients with UUC [72]. More recently, characteristic A:T to T:A transversion mutations were also found in loci of other genes by whole-genome and exome sequencing analysing AA-associated UUC [74,75] illustrating that next-generation sequencing provides a powerful approach to study AA exposure in cohorts or cancers not yet linked to AA.

The A:T to T:A transversions produced after AA exposure are almost exclusively located on the non-transcribed strand of DNA [76] suggesting that this marked strand bias might be linked to the slow removal of dA-AAI adducts from the transcribed strand by transcription-coupled nucleotide excision repair [59,76,77]. Resistance of dA-AAI adducts to global genomic repair may reflect the inability of XPC-RAD23B to recognise and bind to these lesions in duplex DNA [76]. This failure of global genomic repair to excise AA-derived DNA adducts may also account for the persistence of these lesions in human tissues [24]. Indeed this conclusion is in accordance with the detection of AA-DNA adducts (i.e., dA-AAI) in CHN/AAN patients even decades after exposure to AA [53].

All these studies summarised above provided important advances in explaining the molecular mechanism of AA-induced carcinogenesis. As a result, the National Toxicology Program (NTP) [78] lists AA as carcinogenic to humans. The NTP report postulated that “sufficient” scientific evidence is available to conclude that exposure to AA causes urothelial cancer in humans through formation of DNA adducts (specifically, through binding of the reactive metabolite with adenine) and the resulting transversion mutations in oncogenes and the tumour suppressor gene *TP53*. Likewise, AA was classified as human carcinogen (Group 1) by the IARC acting by a genotoxic mechanism [29].

#### 2.1.2. Mechanisms of Enzymatic Activation of AA to Metabolites Forming AA-Derived DNA Adducts and Its Detoxification Resulting in Attenuation of AA-Mediated Diseases

Even though exposure to AA causes the development of UUC associated with AAN/BEN, there are several questions that remained to be answered. Why do only 10–20% of patients in the slimming clinic in Brussels developed AAN [35]? Similarly, only 5–10% of the residents in endemic areas develop BEN [84,85]. In the case of BEN this cannot be attributed easily to preferential exposure of such a small group of the population to AA, but it could result also from other factors. Besides the route of AA exposure and its dose, metabolism determines the biological effective concentration of AA in exposed individuals, which can dictate the development of AAN/BEN and disease progression (i.e., urothelial malignancy). Several endogenous factors might also contribute to disease development, e.g., the efficiencies of bioactivation and/or detoxification of AA, the expression levels of biotransformation enzymes participating in AA metabolism and their activities as well as their genetic and phenotypic polymorphisms.

There are two pathways of AA metabolism that are important for the development of UUC-associated with AAN/BEN: (i) activation of AA to genotoxic intermediates generating DNA adducts; and (ii) detoxification of AA resulting in a decrease in the actual AA concentrations that can lead to the attenuation of nephropathy. The major metabolites of AAI and AAII found in urine and faeces of several animal models and of humans are the aristolactams I and II (for structures of the aristolactams, see Figure 2) [86,87,88]. Other minor metabolites formed through *O*-demethylation (i.e., the formation of 8-hydroxyaristolochic acid I (aristolochic acid Ia, AAIa)) and denitration have also been reported [86,87,88]. The only metabolites identified in humans so far are the aristolactams I and II found in urine [86].

AAI might directly cause interstitial nephropathy while enzymatic activation of AAI to intermediates capable of binding to DNA is a necessary reaction leading to formation of AA-DNA adducts that initiate malignant transformation. Both oxidative and reductive metabolites of AAI are formed in vivo after AAI exposure and are excreted in urine and faeces (reviewed in [16,18,19]).

**Figure 3:**
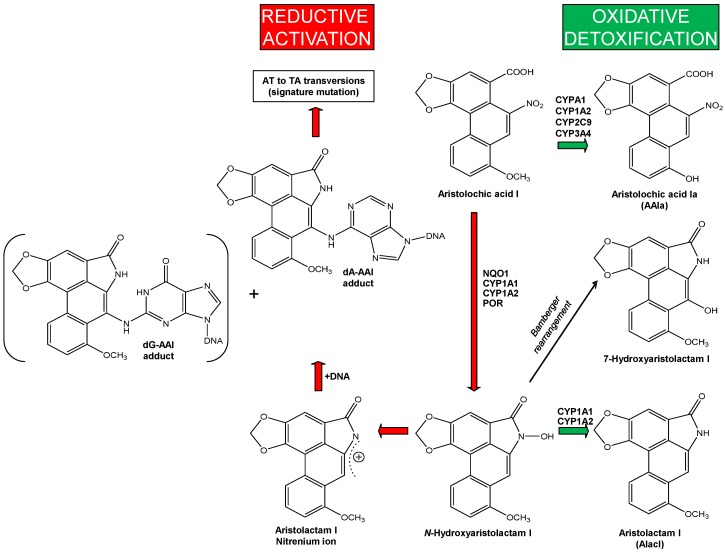
Activation and detoxification pathways of AAI. dA-AAI, 7-(deoxyadenosin-*N*^6^-yl)aristolactam I; dG-AAI, 7-(deoxyguanosin-*N*^2^-yl)aristolactam I; CYP, cytochrome P450; NQO1, NAD(P)H:quinone oxidoreductase; POR, NADPH:CYP oxidoreductase.

Formation of *N*-hydroxyaristolactam I is mediated by reduction of AAI and this metabolite is either further reduced to aristolactam I or rearranged to 7-hydroxyaristolactam I (Figure 3) [87,88]. Aristolactam Ia is one additional AAI metabolite, which is predominantly formed in animal models [87,88] and might be generated either from demethylation of aristolactam I or by reduction of AAIa [89]. Oxidation of AAI leads to AAIa, which is the product of oxidative *O*-demethylation of AAI and considered a detoxification metabolite [21,22,90] (Figure 3). Conversion of *N*-hydroxyaristolactam I to the 7-hydroxyaristolactam I or further reduction to aristolactam I is considered as detoxification pathway, because both metabolites are excreted [87,88]. Moreover, no DNA adducts are generated from AAIa and 7-hydroxyaristolactam I in humans or in animal models (reviewed in [21,22]); essentially no histological changes were determined in kidney of mice treated with AAIa [90]. In the case of aristolactam I, treatment of rats led to ~50-fold lower levels of AAI-DNA adducts (i.e., dA-AAI and dG-AAI) in the kidney than after AAI treatment [91].

**Figure 4:**
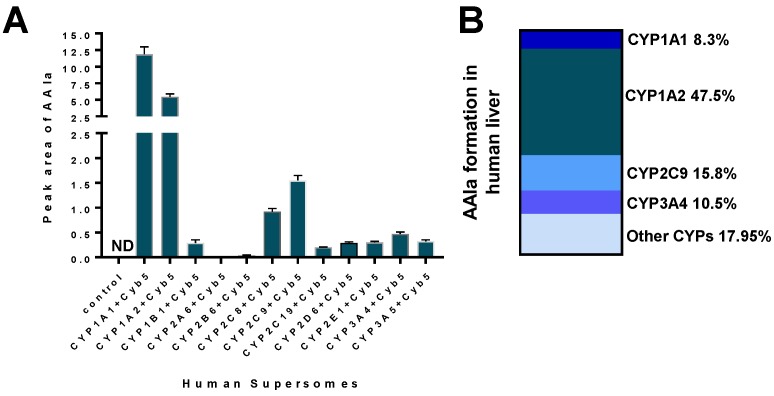
AAIa formation by human recombinant CYP enzymes in the presence of cytochrome *b_5_* (Cyb5) (**A**); Data shown are mean ± SD (*n* = 3). ND, not detected. Data previously published in [92,95,96] (**A**); Contributions of CYP enzymes to AAIa formation in human livers (**B**).

Human cytochrome P450 (CYP) enzymes CYP1A1 and 1A2 are the predominant enzymes oxidising AAI to AAIa under aerobic (i.e., oxidative) conditions [92,93,94,95,96,97]. Of other CYPs, CYP2C (i.e., CYP2C8/9/19), CYP3A (i.e., CYP3A4/5), 2D6, 2E1 and 1B1 also form AAIa, but their efficiency in catalysing this reaction is more than one order of magnitude lower compared to CYP1A enzymes [92,95,96] (Figure 4A). In the liver human CYP1A2 followed by CYP2C9, CYP3A4 and CYP1A1 are the major enzymes contributing to AAI oxidation (Figure 4B) [96].

The mechanism for differing efficacies of these CYPs found experimentally was explained by molecular modeling [96]. The major reasons for the differences observed are based mainly on the finding that CYP2C9 and 3A4 enzymes bind the AAI molecule with a significantly lower affinity and with less suitable orientation than the enzymes of the CYP1A subfamily [96].

The significance of CYP1A1 and 1A2 enzymes to oxidise AAI to AAIa in vivo was demonstrated using *Cyp1a*-knockout and *CYP1A*-humanised mouse lines [92,94,97]. Additional studies were performed in mice and rats where Cyp1a/CYP1A enzymes were enhanced by inducers [98,99]. It was shown that murine Cyp1a1 and 1a2 oxidise AAI to AAIa and protect these animals from AAI-induced acute renal injury [94,100]. Nephrotoxic effects were higher in AAI-treated mice lacking Cyp1a compared to wild-type [94]. Moreover, induction of CYP1A1 and 1A2 in rats resulted in an increase in AAI detoxification to AAIa, thereby reducing the actual concentration of AAI available for reductive activation [98]. More importantly, AAI oxidation to AAIa by human CYP1A1 and 1A2 was also shown in vivo using *CYP1A*-humanised mouse lines [92].

However, a recent study demonstrated that AAII is oxidised to a much lower extent (if any) than AAI; essentially no AAIa or other oxidation products of AAII were found to be formed by microsomal CYP enzymes in vitro [101]. Likewise, in rats exposed to AAII in vivo, no formation of AAIa from AAII was observed [Stiborova et al., unpublished results]. This phenomenon suggests that AAII is metabolised in organisms solely by reductive activation leading to the formation of AAII-DNA adducts [18,20,21]. Interestingly, treatment of rats either with an artificial mixture of AAII and AAI or the natural plant extract containing both AAI and AAII elevated the formation of not only AAII-, but also AAI-derived DNA adducts compared to rats treated with the compounds alone [102]. However, the mechanism of this increase is not yet known and remains to be investigated.

Initial reduction of AAI or AAII to the corresponding *N*-hydroxyaristolactams is the activation pathway responsible for their genotoxic effects. As mentioned above, during this reaction AAI or AAII are enzymatically reduced to cyclic acylnitrenium ions that are capable of binding to the exocyclic amino groups of adenine and guanine in DNA (i.e., dA-AAI, dG-AAI, dA-AAII or dG-AAII) (Figure 2) [42,43,44,45,46,62]. Comparing AAI to AAII, significantly higher levels of AAI-derived DNA adducts than adducts derived from AAII were found in rats and mice in vivo [43,46,103,104,105] and in various enzymatic systems in vitro [45,106,107,108,109,110,111]. However, in C3H/He mice exposed to equivalent doses of AAI and AAII, lower levels of AAII-derived DNA adducts were found only in non-target organs such as liver, stomach, intestine, and lung, in contrast to the primary target tissues such as renal cortex, medulla and bladder (urothelial cells) where the same extent of DNA adducts was found [104]. The apparent discrepancies among the studies [103,104,105] might be attributed to several reasons such as the use of various animal models, utilisation of different treatment protocols and/or employing different methods for the detection of AA-DNA adducts. Nevertheless, differences in the levels of AAI- and AAII-derived DNA adducts might also result from a different enzymatic conversion of these carcinogens (i.e., activation and detoxification). Indeed, several studies showing that AAII is a poorer substrate of the biotransformation enzymes in vitro than AAI support this conclusion [101,106,107,108,109,110].

Several human enzymes capable of activating AAI or AAII by nitroreduction have been identified. Of them, cytosolic NAD(P)H:quinone oxidoreductase (NQO1) was found to be the major cytosolic reductase activating AA both in vitro and in vivo [18,19,20,109,110,111,112,113,114,115,116], while cytosolic xanthine oxidase plays a minor role [20,109,112,114]. The studies on the mechanism of AAI and AAII nitroreduction by NQO1 indicated that direct transfers of electrons from NADPH is mediated through the isoalloxazine ring of its reduced flavine prosthetic group (FAD) [47,110,115]. Moreover, hydroxylic groups of amino acids Tyr128 and Tyr126 present in the NQO1 active site also contribute to AAI reduction; they stabilise the AAI or AAII binding orientation in the NQO1 active site through hydrogen bonding of oxygens of the nitro group [47,110,115].

Studies of several laboratories examining the role of phase II conjugation enzymes in the formation of AAI- and AAII-DNA adducts showed controversial results [47,105,110,112,117,118,119]. No contribution of sulfotransferases (SULT), such as SULT1A, SULT1A3, SULT2E1, SULT2A1, and *N*,*O*-acetyltransferases (NATs) in the bioactivation of AAI and AAII was found in enzymatic cell-free systems in vitro [47,110,112]. This also corresponded to results using human hepatic cytosolic fractions where only NQO1 activity correlated with higher AAI-DNA adduct formation [112]. A recent study utilising transgenic mice carrying a functional human *SULT1A1-SULT1A2* gene cluster or mice with *Sult1a1* knockout showed that conjugation with the active sulfate (i.e., 3′-phosphoadenosine-5′-phosphosulfate [PAPS]) catalysed by human SULT1A1 and murine Sult1a1 do not contribute to bioactivation of AAI and AAII in vivo [105]. In contrast, Meinl et al. [119] showed that expression of human SULT1A1 in bacterial and mammalian cells increased the mutagenicity AA. Sidorenko et al. [117] found that *O*-sulfonated and *O*-acetylated *N*-hydroxyaristolactam I and II form DNA adducts in vitro and that binding of *N*-hydroxyaristolactam I and II to DNA was stimulated by mouse cytosol in the presence of the cofactor PAPS [112]. Moreover, human SULT1B1, SULT1A1 and SULT1A2 were capable of stimulating DNA adduct formation by *N*-hydroxyaristolactam I and II [117]. Likewise, Hashimoto et al. [118] showed that activation of AAI and *N*-hydroxyaristolactam I is potentiated by SULT1A1 in human HK-2 kidney and skin fibroblast GM00637 cells. Consequently, further studies are necessary to resolve the reasons responsible for these discrepancies, for example whether different experimental approaches used in these studies might be the reason.

**Figure 5:**
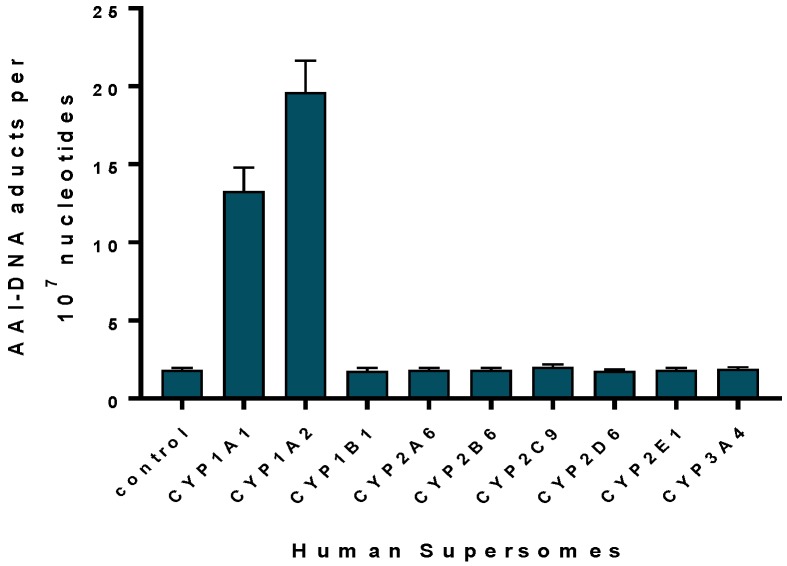
AAI-DNA adduct formation by human CYP enzymes. Control, the POR enzyme was only present adapted from [115,120].

Human microsomal enzymes such as CYPs are also capable of reducing AAI and AAII, while NADPH:CYP oxidoreductase (POR), another microsomal enzyme, plays only a minor role in nitroreduction of these compounds [18,20,21,95,96,106,107,114,120,121,122]. Of human enzymes, CYP1A1 and 1A2 are the only enzymes that are able to efficiently activate AAI (Figure 5) and AAII [106] by nitroreduction under anaerobic conditions, while other CYPs are almost ineffective in catalysing this reaction. In contrast to CYP1A1 and 1A2, the closely related CYP1B1, which is lacking the hydroxyl group containing amino acid residues in its active site, is ineffective in catalysing AAI nitroreductase activity (see Figure 5) [120,122,123].

The mechanisms of CYP-mediated reduction, which is a rather rare case for CYP-catalysing reactions [124,125], have been explained for AAI utilising molecular modeling [123] and site-directed mutagenesis studies [122]. The hydroxyl groups of amino acids Ser122/Thr124 in CYP1A1 and 1A2, which are located closed to the nitro group of AAI in the CYP1A1/1A2-AAI binary complexes are necessary for the reaction as they provide the proton required for the stepwise reduction reaction. In contrast, the closely related CYP1B1, which is lacking the hydroxyl group containing residues in its active site (Ala is present instead of Ser or Thr), is ineffective in catalysing AAI nitroreduction [115,122,123]. Participation of CYP1A1 and 1A2 in the reductive activation of AAI was also demonstrated in rodents in vivo. Experimental in vivo models employed in these studies included Hepatic Reductase Null (HRN) [90], *Cyp1a1(−/−)*, *Cyp1a2(−/−)* and *Cyp1a1/1a2(−/−)* [94,97] mouse lines. *CYP1A*-humanised mouse lines that carried functional human *CYP1A1* and *CYP1A2* genes and lack the mouse orthologous genes confirmed the importance of human CYP1A1 and CYP1A2 in AAI bioactivation in vivo [92].

The function of CYP1A1 and 1A2 both in the reductive and oxidative metabolism of AAI can be explained by different binding orientations of this compound in the CYP1A1 and 1A2 active sites dependent on concentrations of oxygen. AAI acts as a ligand substrate for these human enzymes, where it is bound to the heme iron instead of molecular oxygen, and is therefore reduced instead of being oxidised during the CYP-mediated reaction cycle [20,122,123]. Under oxidative conditions, AAI is a classical substrate of CYP1A1 and 1A2, being bound to Compound I (the highly reactive CYP intermediate that is responsible for the CYP-mediated oxidations) of these CYP enzymes. During this process one atom of molecular oxygen is used to *O*-demethylate the methoxy group of AAI to generate AAIa [20,115]. The dual role of CYP1A1 and 1A2 (oxidation versus reduction of AAI) is an important phenomenon, because a balance between reductive activation and oxidative detoxification reactions of AAI is considered to be a critical determinant in the development of AAN/BEN.

The identification of the enzymes metabolising AA (mainly NQO1, CYP1A1/2, CYP2C9, and CYP3A4/5,) is the first and essential step in the evaluation of their contribution to different susceptibility of individuals to this carcinogen and nephrotoxin. Expression levels of these enzymes and their activities might depend on various factors such as their basal expression, regulation, induction and/or inhibition [126] as well as their polymorphisms [127]. All of the above-mentioned enzymes are inducible and their expression can be modulated both by exogenous (environmental) chemicals or drugs and by endogenous compounds such as several hormones (reviewed in [126,127,128,129]). Moreover, it should be emphasised that exposure to AA itself can induce and/or inhibit some of these enzyme (e.g., NQO1 and CYP1A1/2) [97,98,130].

In addition to these factors, genetic polymorphisms in *NQO1*, *CYP1A1/2*, *CYP2C9* and *CYP3A4/5* might also impact on an individual’s susceptibility to AA. The role of some genetic polymorphisms of biotransformation enzymes (*NQO1*, *CYP1A1*, *CYP2D6*, *CYP3A4/5*, *NAT1/2*, *glutathione-S-transferase (GST) GSTT1*, *GSTM1*, *GSTP1* and *GSTA1*) has already been examined in BEN/AAN patients (for a review, see [21,127,131,132,133,134,135,136,137,138]). However, studies investigating the associations of genetic polymorphisms of the enzymes metabolising AA and the risk of developing AAN/BEN and UUC have reported controversial results. Therefore, the real impact of these enzymes on disease still remains to be understood. One of the reasons for these controversial findings might result from the fact that investigations only focus on genetic polymorphisms without taking the actual expression levels of the enzymatically active proteins into account. Therefore, analyses of the expression levels of enzymes metabolising AA and their phenotyping in AAN/BEN patients are the challenge to receive more valuable data on determination how individual enzymes metabolising AA really contribute to AA-mediated nephropathy and cancer risk among these patients.

## 3. Conclusions

The data described in this review underline the fact that chronic intoxication of humans with AA, a plant product of *Aristolochia* species, is the main causal agent for the development of AAN/BEN and associated UUC. This conclusion is based on similarities of the pathology of AAN and BEN, the detection of specific AA-derived DNA adducts in renal tissue of the patients suffering from these diseases and the dominance of the A:T to T:A transversion mutations in the cancer genomes of these patients. The formation of AA-DNA adducts should be utilised not only as a biomarker for the assessment of AA exposure and risk of UUC, but also be used in mechanistic investigations studying host factors (e.g., enzymes catalysing AA metabolism or DNA repair pathways that may remove AA-DNA adducts) that are critical determinants in mediating the risk for AA-induced UUC. Moreover, because the distribution of *Aristolochia* species is worldwide and the use of medicinal herbal remedies containing AA is still widespread, AA might be the cause of yet un-recognised nephropathies and UUC.

## Figures and Tables

**Table 1:** DNA adduct levels (dA-AAI) in human renal tissue from individuals exposed to AA.

Country	DNA Adduct Measured in	DNA Adduct Detected	DNA Adduct Level/10^8^ Nucleotides	Method Used for DNA Adduct Detection	Publication Year	Reference
Belgium	Kidney	dA-AAI	7.0–53.0 (*n* = 6)	TLC ^1 32^P-postlabelling	1996, 1997	[44,62]
Belgium	Kidney	dA-AAI	0.1–16.5 (*n* = 38)	TLC ^32^P-postlabelling	2000	[27]
Belgium	Kidney	dA-AAI	2.9–5.0 (*n* = 2)	TLC ^32^P-postlabelling	2001	[61]
Belgium/China	Kidney	dA-AAI	1.8 (*n* = 1)	TLC ^32^P-postlabelling	2001	[79]
UK	Kidney	dA-AAI	3.8 (*n* = 1)	TLC ^32^P-postlabelling	2001, 2004	[48,49]
Croatia	Kidney	dA-AAI	0.56–1.71 (*n* = 2)	TLC ^32^P-postlabelling	2002	[63]
Belgium	Kidney	dA-AAI	8.1 (*n* = 1)	TLC ^32^P-postlabelling	2003	[80]
France	Kidney	dA-AAI	0.1–5.4 (*n* = 2)	TLC ^32^P-postlabelling	2004	[34]
China	Kidney	dA-AAI	Detected, but not quantified	TLC ^32^P-postlabelling	2005	[81]
USA	Kidney (cortex, medulla, and pelvis) Kidney (cortex)	dA-AA dA-AAI	11.0–34 (*n* = 1) Detected, but not quantified (*n* = 1)	PAGE ^2 32^P-postlabelling Mass spectrometry	2007	[64]
Croatia	Kidney	dA-AA	8–59 (*n* = 4)	PAGE ^32^P-postlabelling	2007	[64]
Taiwan	Kidney	dA-AA	1.4–234 (89/148 (60%))	PAGE ^32^P-postlabelling	2012	[72]
Bosnia, Croatia & Serbia	Kidney	dA-AA	0.2–19.2 (47/67 (70%))	PAGE ^32^P-postlabelling	2012	[65]
Romania	Kidney	dA-AAI	0.3–6.5 (*n* = 7)	TLC ^32^P-postlabelling	2012	[30]
Belgium	Kidney Kidney	dA-AAI dA-AAI	2–22 (*n* = 11) Detected, but not quantified (*n* = 1)	TLC ^32^P-postlabelling Mass spectrometry	2014	[53]
Croatia & Serbia	Kidney	dA-AAI	0.2–7.0 (*n* = 15)	Mass spectrometry	2014	[82]
Belgium	Kidney	dA-AAI	5 (*n* = 1)	TLC ^32^P-postlabelling	2015	[83]
Romania	Kidney	dA-AAI	0.7–26.8 (*n* = 14)	Mass spectrometry	2016	[71]
Taiwan	Kidney	dA-AAI	0.3–258 (39/51 (76%))	Mass spectrometry	2016	[31]

^1^ Thin-layer chromatography, ^2^ Polyacrylamide gel electrophoresis.
